# Clinical features of macrophage activation syndrome as the onset manifestation of juvenile systemic lupus erythematosus

**DOI:** 10.1093/rap/rkz013

**Published:** 2019-05-14

**Authors:** Satoshi Sato, Yoji Uejima, Yuki Arakawa, Mihoko Furuichi, Eisuke Suganuma, Shuichiro Fujinaga, Atsuko Nakazawa, Yutaka Kawano

**Affiliations:** 1 Division of Infectious Diseases and Immunology; 2 Division of Hematology/Oncology; 3 Division of Nephrology; 4 Division of Pathology, Saitama Children's Medical Center, Saitama, Japan

**Keywords:** macrophage activation syndrome, systemic lupus erythematosus, hyperferritinaemia, ferritin, haemophagocytic syndrome, haemophagocytic lymphohistiocytosis

## Abstract

**Objectives:**

Macrophage activation syndrome (MAS) is a severe complication of juvenile systemic lupus erythematosus (jSLE). However, little is known about the association between these conditions, especially in terms of MAS as the initial manifestation of jSLE. The aim of this study was to determine the clinical features of MAS as the initial manifestation of jSLE.

**Methods:**

We carried out a retrospective review of the clinical features of MAS cases diagnosed concomitantly with jSLE from 2004 to 2016. Data from these patients were compared with those from a control group consisting of jSLE patients without MAS.

**Results:**

Eleven (23.9%) of the 46 patients recruited for this study were diagnosed with MAS during the initial stage of jSLE. The between-group comparisons demonstrated that fever, leucopenia, hyperferritinaemia and increased aspartate aminotransferase were more frequently observed in jSLE patients with MAS than in controls (*P*<0.01). Importantly, neurological symptoms were significantly more common in patients with MAS than in controls (*P*<0.01), with 6 (54.6%) of the 11 MAS patients affected. For treatment, all 11 patients with both jSLE and MAS were administered CSs upon diagnosis, and 7 received immunosuppressants. No patient involved in this study died.

**Conclusion:**

MAS can develop as the initial manifestation of jSLE. MAS with jSLE should be suspected in patients with fever, hyperferritinaemia, cytopenia and liver disorder. In addition, we found that jSLE patients with MAS had more neurological symptoms than those without. All patients with MAS were successfully treated with CSs. Early diagnosis and intensive therapy are essential in improving clinical outcomes.

**Key messages**

- Macrophage activation syndrome could develop as the initial manifestation of juvenile SLE.
- High fever and hyperferritinaemia may be suggestive of and be useful in the classification of macrophage activation syndrome.
- Early use of CSs, in conjunction with immunosuppression, is effective in juvenile SLE with macrophage activation syndrome.

## Introduction

Macrophage activation syndrome (MAS) is a severe, potentially life-threatening complication of autoimmune diseases. Specifically, it is a secondary hyper-inflammatory condition involving uncontrolled immune activation caused by excessive proliferation of macrophages and lymphocytes and is considered a secondary form of haemophagocytic lymphohistiocytosis (HLH) [1, 2]. Among systemic autoimmune diseases, MAS has been described mainly in association with systemic JIA. However, it has been increasingly recognized as a complication of juvenile SLE (jSLE), with studies showing that the incidence of MAS in jSLE ranges from 5.5 to 9% [3, 4].

The common clinical features that are used to establish a diagnosis of MAS include fever, cytopenia, hepatomegaly, splenomegaly, coagulopathy with hypofibrinogenaemia, hypertriglyceridaemia and hyperferritinaemia [5, 6]. Recently, Parodi *et al.* [7] published preliminary diagnostic guidelines for MAS as a complication of jSLE. These guidelines were shown to be clinically relevant by Borgia *et al.* [4], who found that all 38 patients in their study of clinically diagnosed childhood-onset SLE also met the criteria of Parodi *et al.* [7] . Moreover, they reported that in 68% of patients, jSLE and MAS were diagnosed simultaneously.

Despite these reports, data on MAS as an initial complication of jSLE are still limited. The aim of the present study, therefore, was to identify key clinical and laboratory characteristics of MAS as initial symptoms of jSLE and to compare these features and those of jSLE without MAS. We also aimed to describe the treatment approach for patients diagnosed with MAS as the initial manifestation of jSLE.

## Methods

### Patient recruitment

Ethical committee approval was obtained from The Saitama Children's Medical Center Ethics Committee for this retrospective study. Informed patient consent was not required. We reviewed the medical records of 46 patients diagnosed with juvenile-onset SLE at the Saitama Children's Medical Center from 2004 to 2016. We considered jSLE when disease onset was at <18 years of age. Furthermore, we considered SLE patients with MAS as the initial manifestation when the interval between the diagnosis of SLE and MAS was <14 days and when the patients were not treated with any specific therapy for SLE. All of them fulfilled the 1997 revised ACR classification criteria for SLE and the 2012 Systemic Lupus International Collaborating Clinics criteria [8, 9]. The SLEDAI-2000 (SLEDAI-2K) was used to assess disease activity [10]. Given that there are no universally accepted diagnostic criteria for MAS in SLE, the diagnosis of MAS was based on the 2009 preliminary diagnostic guidelines for MAS as a complication of jSLE [7]. Patients were required to fulfil at least one of the following clinical criteria: fever, hepatomegaly, splenomegaly, haemorrhagic manifestations and CNS dysfunction; and at least two of the following laboratory criteria: cytopenia affecting at least two of the three peripheral blood lineages (i.e. white blood cell count, <4.0 × 10^9^/l; haemoglobin, <9.0 g/dl; and/or platelet count, <150 × 10^8^/l), increased aspartate aminotransferase (AST; >40 U/l), increased lactate dehydrogenase (LDH; >567 U/l), hypofibrinogenaemia (<1.5 mg/dl), hypertriglyceridaemia (>178 mg/dl), hyperferritinaemia (>500 ng/ml) and macrophage haemophagocytosis in the bone marrow. The HLH-2004 diagnostic guidelines were also followed for the diagnosis of MAS [5]. Sixteen patients were excluded because of lack of two or more of the 2009 preliminary diagnostic guidelines and clinical or laboratory criteria; thus, there were 30 patients for our between-group analyses.

SLE patients with no MAS episodes and those who did not meet the MAS diagnostic criteria on admission were selected as the control group for this study. Data from these patients were compared with those from patients diagnosed with MAS in jSLE.

### Variables assessed in between-group analyses

We conducted a retrospective analysis of the following variables: age at the time of diagnosis, gender, clinical features, SLEDAI-2K score, medication received during the acute disease course and prognosis. Clinical jSLE data included in our analyses were the incidence of nephritis, serositis, neurological dysfunction, haematological disorders and signs of active infections (in all cases; blood and urine cultures, serology of EBV and CMV). Laboratory findings that were evaluated included white blood cell, haemoglobin and platelet counts; ESR; levels of glutamate oxaloacetate transaminase, creatine phosphokinase, LDH, CRP, ferritin, fibrinogen, triglycerides, complement 3 (C3), complement 4 (C4), fibrin degradation product, D-dimer, ANA, anti-dsDNA antibodies, anti-RNP antibodies, anti-Smith antigen (Sm) antibody, anti-SSA antibodies and anti-SSB antibodies; and serum soluble IL-2 receptor (sIL-2R) using an enzyme-linked immunosorbent assay, NK cell activity and evidence of macrophage haemophagocytosis in aspirated bone marrow or lymph node biopsy samples.

### Statistical analysis

Statistical analysis was performed using Fisher's exact test, the χ^2^ test, the Mann–Whitney *U*-test and Student's *t*-test as appropriate for between-group comparisons. A value of *P*<0.05 was considered statistically significant.

## Results

### Patient demographics and clinical characteristics

During the study period, 46 new-onset jSLE patients were admitted to our hospital. Eleven (23.9%) jSLE patients concomitantly satisfied the 2009 preliminary diagnostic guidelines for MAS at admission (Table 1). Furthermore, 7 (63.6%) of the 11 patients met the HLH-2004 diagnostic criteria, whereas 1 patient (no. 1) did not. The remaining 3 patients (nos 3, 8 and 11) lacked data, such as serum ferritin and sIL-2R levels, NK cell activity and degree of haemophagocytosis required for diagnosis using the HLH-2004 criteria.

**Table 1:** Summary of clinical and laboratory features, complications and treatment of patients with macrophage activation syndrome in juvenile SLE

No.	Age	Sex	Fever	Splenomegaly/ hepatomegaly	Cytopenias (WBC /Hb /PLT)		AST (IU/l)	LDH (IU/l)	Fibrinogen (mg/dl)	TGs (mg/dl)	Ferritin (ng/ml)	Haemophagocytosis	sIL-2R (pg/ml)	Low NK cell function	Major complications	Treatment
1	12	F	Y	N/N	N	1600 / 10.1 /16.5	87	518	263	112	927	ND	2240	ND	Anxiety disorder	Glucocorticoid, MMF
2	7	F	Y	N/N	Y	7500 / 5.0 /2.3	134	719	111	231	1401	ND	2595	ND	Seizure disorders, nephritis, APS, Evans syndrome	Glucocorticoid, MPT, IVIG, i.v. CYC
3	12	M	Y	Y/Y	Y	2400 / 10.1 /12.3	108	425	191	108	ND	ND	3294	Y	Nephritis	Glucocorticoid, MPT, MMF
4	13	F	Y	N/N	Y	3300 / 9.6 /12.1	815	1617	118	124	10115	ND	2807	ND	Pancreatitis	Glucocorticoid, MPT, MMF
5	13	M	Y	Y/N	Y	1000 / 7.0 /12.9	369	1687	190	283	14760	Y-BM	1990	Y	Demyelinating syndrome, APS	Glucocorticoid, MPT, i.v. CYC
6	14	F	Y	Y/N	Y	500 / 11.3 /9.8	194	1110	225	203	2232	Y-BM	1254	ND	Lupus enteritis, APS	Glucocorticoid, IVIG
7	12	F	Y	Y/Y	Y	3200 / 7.4 /7.7	54	690	282	148	1127	Y-Lym	4170	N	Pleuritis and pericarditis, APS	Glucocorticoid, AZA
8	14	F	Y	N/N	N	800 / 10.8 /15.9	179	829	211	152	1780	ND	3616	Y	–	Glucocorticoid
9	17	F	Y	N/N	Y	1000 / 9.1 /7.1	156	1151	212	219	5213	ND	2384	ND	Myelopathy	Glucocorticoid, MPT, MMF
10	7	F	Y	N/N	Y	1000 / 10.6 /5.9	172	706	55	136	1245	ND	4107	ND	Psychosis, nephritis	Glucocorticoid, MPT
11	13	F	Y	N/N	Y	1700 / 7.3 /5.3	780	3220	207	170	6940	Y-BM	ND	ND	Cognitive dysfunction	Glucocorticoid

AST: aspartate aminotransferase; F: female; Hb: haemoglobin; LDH: lactate dehydrogenase; M: male; ND: not done; MPT: pulse methylprednisolone therapy; PLT: platelet count; sIL-2R: soluble IL-2 receptor; TGs: triglycerides; WBC: white blood cell.

The median age at which jSLE with MAS was diagnosed was 13.0 ± 2.9 years (range, 7–17 years), with a female:male ratio of 9:2. All patients presented with fever at diagnosis, and splenomegaly was recorded in 36.4% (4/11) and hepatomegaly in 18.2% (2/11) of patients. Laboratory findings at the time of MAS onset included hyperferritinaemia (median, 2006 ng/ml; range, 927–14 760 ng/ml) and increased levels of sIL-2R [median, 2701 U/ml; range, 1254–4170 pg/ml (reference <496 pg/ml)], AST and LDH. NK cell activity was also tested in four patients, with three showing a decrease. In addition, 90.9, 36.4 and 81.8% of the patients developed leucopenia, anaemia and thrombocytopenia, respectively, and only 27.2 and 36.4% had hypofibrinogenaemia and hypertriglyceridaemia, respectively. A bone marrow aspirate or lymph node biopsy was obtained from four patients, all of whom showed haemophagocytosis.

### Between-group comparisons

Our comparison of clinical and laboratory findings at the time of diagnosis between jSLE patients with and without MAS is reported in Table 2. Although fever and splenomegaly were more frequent in patients with than without MAS, there were no between-group differences in the incidence of hepatomegaly and haemorrhage. Significantly higher activated PTT, in addition to higher levels of AST, LDH, fibrinogen and ferritin, were found in jSLE patients with MAS, whereas fibrinogen levels and white blood cell, neutrophil and platelet counts were markedly lower. There were no between-group differences in age at the time of diagnosis, gender, SLEDAI-2K score or the incidence of nephritis. There were also no between-group differences in the levels of sIL-2R; anti-dsDNA, anti-Sm, anti-SSB and aCL antibodies; C3 and C4; ESR; and CRP. Finally, the incidence of neurological symptoms was higher in patients with MAS than in those without MAS. MRI abnormalities were detected in three patients, including neurological involvement with stroke (no. 2), demyelinating syndrome (no. 5) and myelopathy (no. 9).

**Table 2:** Demographic data, clinical manifestations and laboratory findings in 30 juvenile SLE patients with and without macrophage activation syndrome

	With MAS (*n* = 11)	Without MAS (*n* = 19)	*P*-value
At the age of onset of SLE (years)	13.0 (7–17)	12 (8–16)	0.4
Female gender [*n* (%)]	9 (81.9)	17 (89.5)	0.5
Fever [*n* (%)]	11 (100)	9 (47.4)	<0.01
Hepatomegaly [*n* (%)]	2 (18.2)	4 (21.1)	0.6
Splenomegaly [*n* (%)]	4 (36.4)	1 (5.3)	<0.05
Haemorrhage [*n* (%)]	1 (9.1)	0 (0)	0.8
WBC count (/μl)	1600 (500–7500)	3600 (2000–20 000)	<0.01
Neutrophil count (/μl)	1100 (140–5300)	2140 (600–17 680)	<0.01
Haemoglobin (g/dl)	9.6 (5–11.3)	10.5 (4.1–13.1)	0.06
Platelet count (× 10^8^/l)	9.8 (2.3–16.5)	16.9 (0.2–23.4)	<0.05
AST (U/l)	172 (54–815)	26 (15–133)	<0.01
LDH (U/l)	829 (425–3220)	273 (150–755)	<0.01
Fibrinogen (mg/dl)	199 (55–282)	281 (217–678) (*n* = 18)	<0.01
Triglycerides (mg/dl)	152 (108–283)	125 (47–248)	0.1
Ferritin (ng/ml)	2006 (927–14 760) (*n*=10)	145 (5.6–482) (*n* = 17)	<0.01
sIL-2R (U/ml)	2701 (1254–4170) (*n*=10)	1248 (554–3776) (*n* = 9)	0.7
Positive ANA [*n* (%)]	11 (100)	18 (94.7)	0.6
Positive anti-dsDNA [*n* (%)]	10 (90.9)	17 (89.5)	0.7
Positive anti-SM [*n* (%)]	6 (54.5)	12 (63.2)	0.5
Positive anti-RNP [*n* (%)]	3 (42.9) (*n*=7)	10 (58.8) (*n* = 17)	0.4
Positive anti-SSA [*n* (%)]	9 (81.8)	10 (52.6)	0.1
Positive anti-SSB [*n* (%)]	4 (36.4)	5 (26.3)	0.8
Positive aCL (IgG) [*n* (%)]	3 (33.3) (*n*=9)	7 (36.8)	0.6
Complement 3 (mg/dl)	40.0 (14–80)	46.5 (16–107)	0.15
Complement 4 mg/dl)	9.0 (2–11)	6.0 (1–27)	0.4
ESR (mm/h)	58 (16–143)	69 (11–125)	0.7
CRP (mg/dl)	0.46 (0.01–3.87)	0.26 (0.01–12.5)	0.4
Prothrombin time (s)	12.6 (10.9–14.4)	12.5 (10.3–21.3)	0.7
Activated partial thromboplastin time (s)	39.0 (27.5–63.9)	31.2 (26.8–81.5)	<0.05
Fibrin degradation product (μg/dl)	8.6 (1.6–86.6)	4.4 (0.9–14.4) (*n* = 17)	0.1
D-dimer (μg/dl)	4.9 (1.3–39.1) (*n*=10)	2.4 (0.5–6.7) (*n* = 17)	0.1
SLEDAI-2K	16 (8–43)	13 (6–25)	0.2
Neurological dysfunction [*n* (%)]	6 (54.5)	1 (5.3)	<0.01
Nephritis	3 (27.3)	9 (47.4)	0.2

Data are reported as *n* (range) unless stated otherwise. AST: aspartate aminotransferase; LDH: lactate dehydrogenase; MAS: macrophage activation syndrome; sIL-2R: soluble IL-2 receptor; WBC: white blood cells.

### Patient treatment and outcomes

All patients received CSs once a diagnosis of jSLE with MAS was established, with six receiving pulsed methylprednisolone therapy. Furthermore, two patients were treated with IVIG and seven with immunosuppressants including CVC, MMF and AZA. No between-group difference was noted in the treatments received (data not shown), and no patients involved in this study died. Five of six MAS patients who had neurological manifestations showed gradual improvement in their neurological symptoms; one patient retained a mild anxiety disorder.

## Discussion

In this study, we report data from 11 patients with juvenile-onset SLE complicated with MAS at a single children’s medical centre. Although other studies have reported that the incidence of MAS in jSLE ranges from 5.5 to 9%, the prevalence of MAS was higher in our study at 23.9% [3, 4]. This can be accounted for by exclusion of patients who were already undergoing treatment for jSLE at other hospitals. In addition, jSLE cases severely complicated with MAS are referred to our hospital because it is the sole tertiary hospital for paediatric rheumatology in a high-population area.

MAS is recognized as a life-threatening complication not only of systemic onset JIA, but also of jSLE [11]. Diagnosis of MAS is usually challenging, because there is no single clinical or laboratory parameter that can be used to aid in its diagnosis, and haemophagocytosis may not always be seen in MAS patients [12, 13]. Furthermore, the features of jSLE complicated with MAS may be difficult to distinguish from or overlapping those of other conditions that may present in a similar manner, such as ITP, autoimmune haemolytic anaemia, APS, thrombotic thrombocytopenic purpura, CNS dysfunction, liver disease and systemic infections [14]. To overcome these difficulties, Parodi *et al.* [7] developed their 2009 preliminary diagnostic guidelines for MAS as a complication of jSLE, which were shown by Borgia *et al.* [4] to be clinically relevant. We found further evidence for the clinical significance of the guidelines of Parodi *et al.* [7], finding that 7 (63.6%) of the 11 patients diagnosed with MAS using these guidelines also met the HLH-2004 diagnostic criteria. However, it should be noted that one patient (no. 1) did not meet these criteria, and the remaining three patients (nos 3, 8 and 11) lacked sufficient clinical data. Among the clinical and laboratory features of jSLE with MAS identified by the guidelines by Parodi *et al.* [7], fever was the most frequent symptom in the present study. This is in accordance with other studies, in which fever is the mainstay manifestation of MAS. Other abnormal findings that are known to occur in MAS are severe cytopenia and hepatic dysfunction. Accordingly, we found both splenomegaly and hepatomegaly in MAS patients, although the former was more common in this group. We also found greater frequencies of leucopenia, neutropenia and thrombocytopenia in patients with MAS than in those without. In addition, decreased NK cell activity and increased sIL-2R have been recognized as diagnostic features of MAS. However, these tests are generally not used for MAS diagnosis in jSLE owing to their lack of recognition, although we did find decreased NK cell activity in three of the four patients tested. As such, the diagnosis of MAS remains largely dependent on clinical manifestations and serum ferritin levels. Finally, six of the MAS patients showed CNS manifestations, though it is difficult to determine whether CNS dysfunction can be attributed to MAS or to active SLE [4, 15]. Nevertheless, our results corroborate those of an earlier study on jSLE with MAS, in which a higher frequency of CNS involvement was found in patients with MAS than in those without [7, 11].

Regarding the development of MAS in jSLE, it has been thought that MAS is a rare complication of jSLE, and occurs especially at jSLE onset. It is also widely recognized that infection and active disease are the main trigger factors for MAS [16, 17]. However, we found no specific infectious agents in blood or urine, and there was no between-group difference in CRP level. The median SLEDAI-2K score in jSLE patients with MAS was 16 (8–43), which reflects high jSLE disease activity. However, SLEDAI-2K scores did not differ between the two groups; as such, it remains unclear whether jSLE activity itself is responsible for excessive macrophage activation.

In terms of treatment, the therapeutic strategy for jSLE with MAS has not yet been established. The mainstay of MAS treatment is a high dose of CSs as first-line therapy. Immunosuppressive agents, including CYC, MMF, rituximab, anakinra, etoposide, IVIG and plasma exchange, may be added in severe or refractory cases of MAS with SLE, [4, 6, 18, 19]. In the present study, all patients were promptly treated with a high dose of CSs, including an i.v. pulsed methylprednisolone therapy upon diagnosis, and the combination of CSs with other immunosuppressive therapies (frequently, MMF and CYC) in the severe cases of SLE was effective, with no patients dying as a result of MAS. In another study, however, a mortality rate of 5% among 38 cases of jSLE-associated MAS was reported, indicating that this may be a potentially fatal condition [4].

Despite the insights provided by the present study, it does have some limitations. First, this is a single-centre study assessing data from a small number of patients diagnosed and managed by local practices. Patients referred to a tertiary children's hospital may have more advanced or refractory conditions, and thus may not be representative of the general diseased population. Use of the 2009 preliminary diagnostic criteria for MAS may be considered more lenient than the 2004 HLH diagnostic criteria and may have led to overdiagnosis in this cohort. Second, blood tests for serum ferritin and sIL-2R levels, NK cell activity and haemophagocytosis in bone marrow were not conducted for all patients. Further studies are therefore needed to establish more robustly the clinical relevance of factors used to diagnose MAS in jSLE.

Nevertheless, MAS is a severe complication and may develop as the initial manifestation of jSLE. The physician must consider treatment of the underlying clinical condition and laboratory features of MAS at the early active stage of SLE. We demonstrated that a feasible screening for MAS should include monitoring of serum ferritin levels and assessment of neurological symptoms. The 2009 preliminary diagnostic guidelines for MAS with jSLE helped with the classification of patients suffering from these conditions in the present study. Lastly, a good prognosis can be obtained with early diagnosis and prompt treatment, which is important, considering the life-threatening potential of early-stage jSLE with MAS.

*Funding*: No specific funding was received from any funding bodies in the public, commercial or not-for-profit sectors to carry out the work described in this manuscript.

*Disclosure statement*: The authors have declared no conflicts of interest.
